# Self-assembly of Pseudo-Dipolar Nanoparticles at Low Densities and Strong Coupling

**DOI:** 10.1038/s41598-020-60417-4

**Published:** 2020-03-04

**Authors:** Mariano E. Brito, Marcelo A. Carignano, Verónica I. Marconi

**Affiliations:** 1 Institute of Complex Systems (ICS-3), Forschungszentrum Jülich, D-52425 Jülich, Germany; 2 Facultad de Matemática, Astronomía, Física y Computación, Universidad Nacional de Córdoba, X5000HUA Córdoba, Argentina; 3 Qatar Environment and Energy Research Institute, Hamad Bin Khalifa University, P.O. Box 34110 Doha, Qatar; 4 IFEG-CONICET, X5000HUA, Córdoba, Argentina

**Keywords:** Colloids, Coarse-grained models

## Abstract

Nanocolloids having directional interactions are highly relevant for designing new self-assembled materials easy to control. In this article we report stochastic dynamics simulations of finite-size pseudo-dipolar colloids immersed in an implicit dielectric solvent using a realistic continuous description of the quasi-hard Coulombic interaction. We investigate structural and dynamical properties near the low-temperature and highly-diluted limits. This system self-assembles in a rich variety of string-like configurations, depicting three clearly distinguishable regimes with decreasing temperature: *fluid*, composed by isolated colloids; *string-fluid*, a gas of short string-like clusters; and *string-gel*, a percolated network. By structural characterization using radial distribution functions and cluster properties, we calculate the state diagram, verifying the presence of string-fluid regime. Regarding the string-gel regime, we show that the antiparallel alignment of the network chains arises as a novel self-assembly mechanism when the characteristic interaction energy exceeds the thermal energy in two orders of magnitude, *u*_*d*_/*k*_*B*_*T* ≈ 100. This is associated to relevant structural modifications in the network connectivity and porosity. Furthermore, our results give insights about the dynamically-arrested nature of the string-gel regime, where we show that the slow relaxation takes place in minuscule energy steps that reflect local rearrangements of the network.

## Introduction

Colloidal particles with directional interactions, so called patchy particles, are increasingly attracting the attention of many scientists since they represent a building block for the programmable design of complex structured self-assembled materials^1–5^. Recent advances in the fabrication of colloidal particles with anisotropic surface charge triggered theoretical efforts, aimed to understand the relation between particle concentration and interaction strength, and how these factors determine the structure of the system.

One of the most-relevant theoretical models, used to describe dipolar fluids, is the Dipolar Hard-Sphere model (DHS), which has been widely studied during the last years. It consists of a point-like dipole embedded in the center of a hard sphere, and it is particularly relevant to theoretical studies since it is the simplest model that incorporates anisotropic interactions. Different studies have focused on describing the phase diagram of this complex system. In a very wide range of concentrations and particularly focused on the crystalline structures, the phase diagrams of hard and soft spheres with a fixed dipole moment have been determined by calculating the Helmholtz free energy using simulations^6^, and the ground states of crystalline structures arising from Stockmayer (i.e., Lennard-Jones plus dipolar) interaction have been studied with Ewald summation^7^. In the dilute regime, it has been theoretically predicted^8^ and experimentally observed^9^ that suspensions of dipolar colloids present string-like aggregates, where the colloids self-assemble into the most-energetically-favorable *tail-nose* configuration. This type of self-assembly leads to different aggregated regimes, being the gel regime the most relevant. Consequently, many works have focused on studying the properties of DHS fluids at low temperature and density, in order to understand and explore this interesting regime and its potential applications. In particular, significant challenges emerge in this region of phase diagram as consequence of the high computational demand on long-range electrostatic interaction calculations and the long relaxation time of the system structure^10^. The structural properties have been analyzed by means of Monte Carlo (MC) simulation techniques, with the aim of characterizing the different regions according to their aggregate topologies, such as percolated networks, strings and rings^11–13^. Special interest has been addressed to understand the role of the topological defects because they not only determine and characterize the regimes appearing at low temperature and density^8^, but also they are responsible of the arising of a rich variety of arrangements in a mesoscopic scale^14,15^.

Other studies have focused on the description of dipolar colloids by means of hard-sphere colloids with an embedded finite dipole. Molecular Dynamics (MD) simulations have been used, where the particle-particle interactions are modeled with a discontinuous potential, to explore the self-assembly, structure, crystallization and gelation of this system, in order to provide a wide description of the phase diagram of both single-specie^16^ and mixture^17^ of dipolar suspensions. Variations of this model with implementation of a continuous interaction potential have been implemented for describing the kinetics of the gel formation^18^, and the influence of the particle elongation in the system percolation and its dynamic arrest^19,20^. An optimized Cluster Move MC algorithm has been also proposed, in order to efficiently explore the highly-diluted regime^21^. The behavior of dipolar fluids has also been investigated under the effect of an external fields. Using a model of charged soft dumbbells in MD simulations inspired in refs. ^19,20^, Ilg and Del Gado studied the properties of the network structure of the dipolar gel and its response to an external electric field^22^. Along these lines, Dussi *et al*.^23^ performed a Monte Carlo study investigating further the role of the dumbbell geometry at low density and moderate coupling, finding that the self-assembly of the particles in ring structures becomes dominant with respect to the formation of chain-like structures.

In this article, based on previous experience with isotropic Lennard-Jones colloidal gels^24^, we explore the state diagram of very-dilute pseudo-dipolar solutions at low temperature distinctively using Stochastic Dynamics (SD) simulations with implicit solvent and a simple continuous model for the pair interaction between colloids. The main advantage of our model is that it provides a more realistic description of both the forces present in the system and its time evolution in this new region of the phase diagram. As we shall demonstrate, we analyze and characterize the different regimes, identifying new structural and dynamical signatures, such as the bundle formation and the step-wise relaxation.

## Model and Method

We consider a solution of quasi-hard colloids with embedded charge distribution having a net dipole moment. This type of particles exhibit anisotropic interactions that drive them to self-assemble into clusters, strings or networks depending on the conditions. Due to the anisotropy, the assembly takes place in preferential configurations. The most-favorable configuration is the tail-nose assembly, which leads to the formation of string-like (chain-like) clusters at low densities. For high electric coupling the string-like clusters may percolate, forming a network.

**Figure 1:**
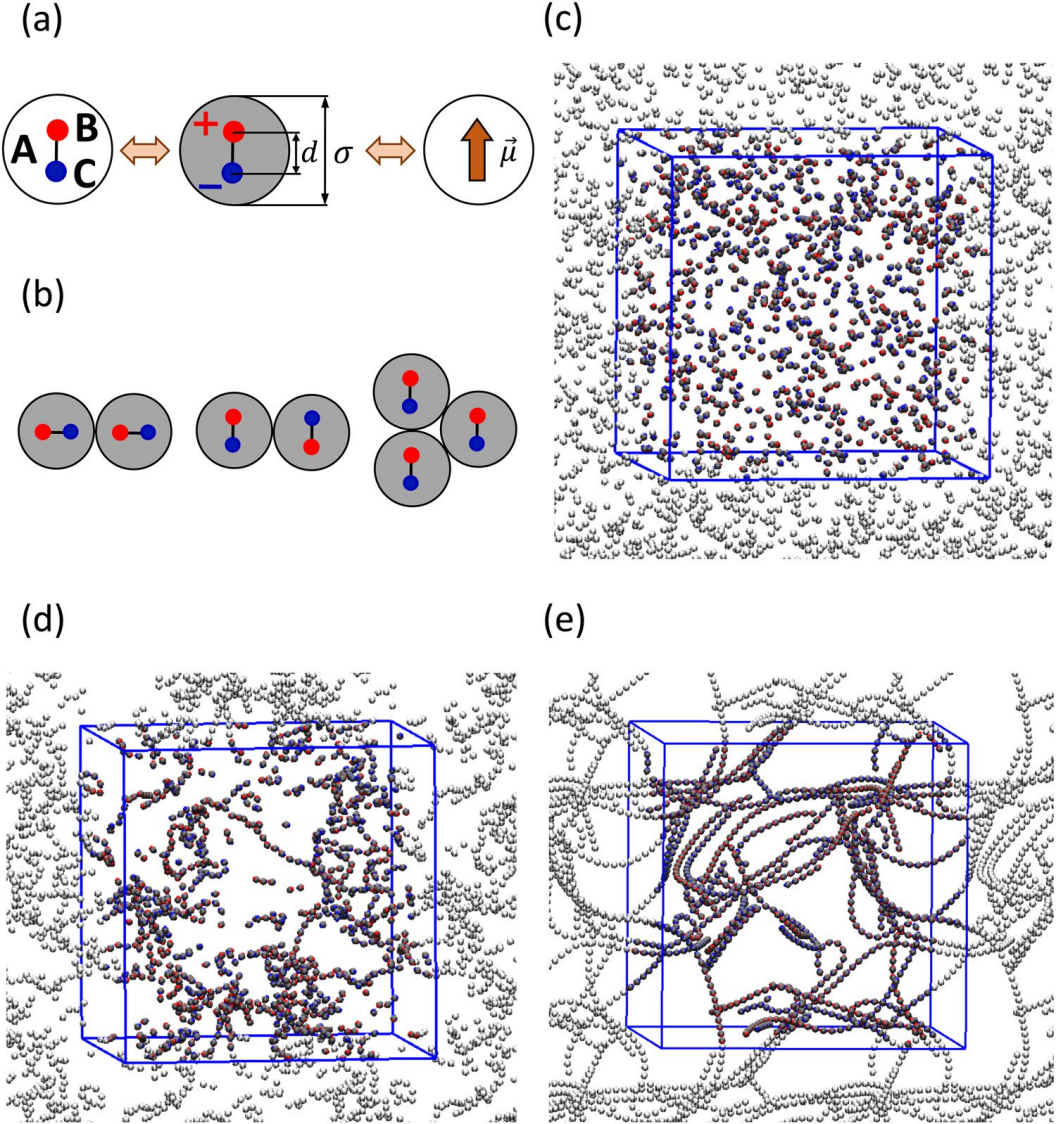
(**a**) Schematic representation of the colloid model with an embedded electric dipolar moment *μ*, implemented with three interacting sites A–C: neutral site A with diameter *σ*, sites B and C with charge + *q* and − *q* respectively. The distance between A,B and A–C is constrained to be *σ*/4, and the distance B,C is constrained to *d* = *σ*/2. (**b**) Examples of colloidal arrangements: (from left to right) tail-nose, antiparallel and triple-point assemblies. Snapshots representative of the (**c**) fluid, (**d**) string-fluid and (**e**) string-gel regimes. Figures (**c**,**e**) generated with VMD^44,45^.

In our simulation model, we mimic pseudo-dipolar colloids with quasi-hard particles containing an explicit electric dipole, namely, the electric dipole is modelled by two point charges separated by a finite distance, instead of a point dipole located at the center of the spherical colloidal particle. We investigate a system of *N* colloids with particle diameter *σ*, contained in a cubic simulation box of volume *V*. Each particle is described by three interaction sites: A, B and C. The site A corresponds to a purely repulsive central interaction potential representing the size of the spherical colloid. The explicit electric pseudo-dipole consists of a pair of point charges +*q* and −*q* located on the sites B and C, respectively, separated a fixed distance *d* that results in a dipole moment *μ* = *q*. *d*. The relative position of the three interacting sites is kept constant using the LINCS algorithm^25^. Fig. 1(a) displays a schematic representation of the colloidal particle model. Since each particle is described by three interaction sites, the simulation involves *N*_p_ = 3*N* particles in total. The interaction energy between two particles is given by

1 $$U(r)={U}_{c}(r)+{U}_{e}(r)$$

2 $$\hspace{4.8pc}=\,{u}_{d}{\left(\frac{\sigma }{r}\right)}^{36}+{\sum }_{i,j}\frac{1}{4\pi {\epsilon }_{0}\epsilon }\frac{{q}_{i}{q}_{j}}{{r}_{ij}},$$

where *r* is the distance between the centers of the particles, and *ε*_0_ and *ε* are the vacuum electric permittivity and the relative permittivity of the solvent, respectively. *u*_*d*_ is a reference unit of energy, *σ* represents the diameter of the colloid and *r*_*ij*_ is the distance between charge *i* and charge *j* in each one of the two particles. The first term on the right-hand side corresponds to the repulsive quasi-hard core interaction and the second term corresponds to the Coulombic interactions. Some previous works on similar systems have used hard spheres to represent the colloidal particles^16^. A hard-sphere interacting potential can be mathematically described by *lim*_*n*→*∞*_(*σ*/*r*)^*n*^. The use of this potential form with *n* = 36 or higher, which has been employed previously by several other authors^26–31^, represents a reasonable approximation to a hard sphere while keeping a continuous form that ensures the safe calculation of the forces between the particles.

We performed SD simulations^32^, a method in which a friction term and thermal noise are added to the classical Newton’s equation of motion:

3 $$m\frac{{d}^{2}{{\bf{r}}}_{i}}{d{t}^{2}}=-m\xi \frac{d{{\bf{r}}}_{i}}{dt}+{{\bf{f}}}_{i}(r)+{{\boldsymbol{\eta }}}_{i}.$$

Here, **r**_*i*_ is the position vector of the particle *i*, *m* is the mass of the particles and *ξ* the friction constant. The first and second terms on the right-hand side represent the friction and the interaction forces respectively. The latter has the form **f**_*i*_(*r*) = −∇ *U* and is computed considering a cut-off distance equal to 4*σ*. The use of a spherical cut-off on dipolar interactions is a common assumption in coarse grained models that does not strongly affect the physics of the model system^16,17,21,33–35^ within the studied range of parameters. The last term in Eq. 3, ***η***, is a white noise satisfying $\langle {\eta }_{i}(t){\eta }_{j}(t+s)\rangle =2m\xi {k}_{B}T\delta (s){\delta }_{ij}$ for each pair of components *ij*. Note that the noise term acts over the three interacting sites allowing for the particle to undergo random translation and rotational displacements. The use of this particular dynamic model is important for two main reasons. First, the random noise mimics the thermal interaction of the particles with the solvent. Second, the random noise has the additional effect of distributing the kinetic energy among all the particles, which is especially important for simulating low concentrations. The main advantage of using SD is that the method leads to a more even partitioning of the kinetic energy. By performing standard MD simulations of diluted systems coupled to a single thermostat, part of the system decreases its temperature as other parts become hotter in such a way that the total kinetic energy reflects the target temperature. This effect could lead to spurious results^18^ that can be prevented using a stochastic method.

All simulations were performed using Gromacs v.4.5.5^36–39^. This program requires to input specific units of the physical parameters, however, all the results will be expressed in terms of reduced units. We define the unit of length as *σ*, which is approximately the size of the particles. The unit of energy is based on the dipole-dipole interaction at the most favorable (antiparallel) orientation, *u*_*d*_ = (1/4*πε*_0_*ε*)(*μ*^2^/*σ*^3^). This allow us to define the reduced potential energy U^*^ = *U*/*u*_*d*_ and the reduced temperature T^*^ = *k*_B_*T*/*u*_*d*_, which gives a measure of the thermal energy against the characteristic electrostatic energy, and characterizes the relative energies where the different regimes emerge. Finally, we define the reduced time ${t}^{\ast }=t/\sqrt{m{\sigma }^{2}/{u}_{d}}$, which is approximately equal to the time for a particle to displace its size under a kinetic energy similar to the characteristic unit of energy *u*_*d*_. Our simulation methodology contrasts with that of previous studies in which a discontinuous dynamic approach was implemented to simulate particles interacting via a three-step square shoulder potential mimicking the dipole-dipole interaction between spheres^16^, and also in the fact that we consider a finite dipole, which is embedded inside a quasi-hard particle^12^. In comparison with models where the electric part is similarly implemented in charged soft dumbbells^19,20,22^, we model spherical colloids with harder short-range repulsion. For distances *d* ≪ *r* < *L*, our model would in principle approach that of a point dipole. However, since we impose a cut-off at a finite distance the long-range nature of the dipolar interactions is not considered. The interaction model that we use is qualitatively akin to the one used by Goyal *et al*.^16^, but it is described in terms of smooth and continuous interactions, similarly to refs. ^19,20,22^, which makes the interaction potential appropriate for numerical derivation. In order to explicitly distinguish our approach from a long-range dipolar interaction potential we refer to our model as *pseudo-dipolar*.

In order to quantify the connectivity of the system, we establish a criteria to decide whether two colloids are bound: particle *i* is bound to particle *j*, if *r*_*ij*_ < *r*_b_, where *r*_*ij*_ is the center-to-center distance between them and *r*_*b*_ is the bond distance threshold. *r*_b_ is defined as the position of the first minimum of the radial distribution function, *g*(*r*). Therefore, the calculation of both *r*_b_ and the system connectivity properties is a post analysis, since *g*(*r*) depends on the concentration and the temperature of the system. The variations in *r*_b_ could be up to 30%, in particular for very low temperature where the system becomes highly structured making the determination of first minimum of *g*(*r*) somewhat ambiguous. Small variations in the election of *r*_b_ might produce noticeable variations in the calculated cluster properties but they do not affect the results concerning regime identification due to the presence of a percolated macrocluster.

## Results

To characterize the behavior of the model system in the different regimes, we have analyzed the potential energy and the structural properties. The system is composed of *N* = 1000 colloids dispersed in a solvent of relative permittivity *ε* and occupying a volume *V*. We define the colloidal volume fraction as *ϕ* = *πNσ*^3^/(6*V*) that takes values on the range 0.0061–0.09. The strength of the inter-particle coupling is controlled by the reduced temperature T^*^, which takes values in the range 8 × 10^−5^ − 0.55. In units of thermal energy this range corresponds to 1.8 − 1.2 × 10^4^, which contains the experimentally-studied energy range in similar systems^9,40^. The friction coefficient has been set to *ξ* = 1/2t^*^, and the total simulation time (in units of t^*^) was of at least ${t}_{\,{\rm{tot}}\,}^{\ast }=5000$ and extended up to 8000 for some cases. All runs were conducted using a time step Δt^*^ = 1 × 10^−4^.

All simulation runs are started from a disordered configuration obtained with a high temperature preliminary run. Once the system is quenched to the target temperature, it evolves by making associations between particles and decreasing the total potential energy accordingly. Depending of the temperature and overall volume fraction, the potential energy reaches a regime in which the variations are just fluctuations over a mean value, or a slow approach towards that regime.

### Potential energy

**Figure 2:**
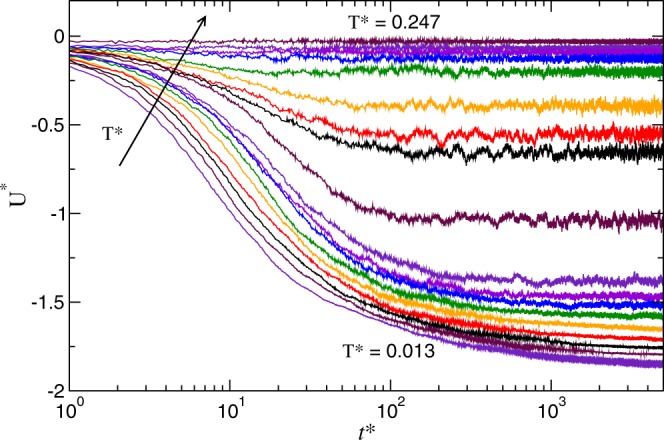
Potential energy U^*^ versus time t^*^ at different temperatures T^*^. Different colors refer to different temperatures in the range 0.013–0.247. All curves correspond to volume fraction *ϕ* = 0.009 and the arrow indicates the direction of increasing T^*^.

At time t^*^ = 0, the system is instantaneously quenched to the target temperature and evolves following the dynamics dictated by Eq. 3. As representative example in Fig. 2, we show the time evolution of the potential energy at fixed volume fraction for different temperatures. At high temperature, there is almost no interaction between the particles and the potential energy remains high. By decreasing the temperature the particles start to form string-like clusters by assembling themselves following the tail-nose configuration, Fig. 1(b). This results in a decrease of the system energy, however retaining its fluid-like character. Further reduction of the temperature results in the formation of a percolating cluster spanning the whole simulation box. These three distinct regimes have been identified in previous works^6,12,16,20,22^ as: *fluid*, *string fluid*, and *string gel*. Fig. 1(c–e) show typical snapshots of these three regimes respectively. Strictly speaking, the two so-called fluid regimes are gas phases in our simulation model, and the string-gel regime could be thought as a (reversible) polymerization of the system involving most of the particles. This polymerization process affects the kinetics of the system, which depends on the particular structure that is formed.

**Figure 3:**
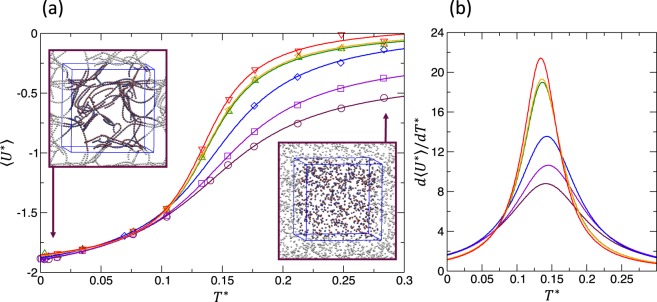
(**a**) Mean potential energy $\left\langle {U}^{\ast }\right\rangle$ as function of the system temperature T^*^. Different colors refer to different volume fractions, *ϕ* = 0.006; 0.0082; 0.0089; 0.019; 0.049; 0.090 (from top to bottom). Solid lines are best fits of the simulation results using ${f}_{U}({T}^{\ast })={A}_{0}+{A}_{1}\arctan ({T}^{\ast }/{A}_{2}-{A}_{3})$. Insets generated with VMD^44,45^. (**b**) Heat capacity calculated from the best fit.

The final potential energy of the system measures the degree of association between particles. The mean final potential energy, $\left\langle {U}^{\ast }\right\rangle$, is obtained by taking time average over the last 1000 time units where the energy remains nearly constant. The temperature dependence of $\left\langle {U}^{\ast }\right\rangle$ for different volume fractions is displayed in Fig. 3. The results from simulations are represented by points and the solid lines are the best fits using ${f}_{U}({T}^{\ast })={A}_{0}+{A}_{1}\arctan ({T}^{\ast }/{A}_{2}-{A}_{3})$. For all cases, the fitting function captures the trends with a correlation coefficient larger than 0.99. All the cases in Fig. 3 suggest a clear regime transition between string-gel regime at low temperature and fluid phase at high temperature, where the energy reaches a low and high plateau, respectively. It is important to note that for the high-temperature regime the system reaches its thermodynamic equilibrium. However, for the lower-temperature case the final state is either a metastable or a particular conformation undergoing a slow evolution toward a stable state. The function *f*_*U*_(T^*^) represents the caloric curve and its derivative the heat capacity that, in the thermodynamics limit, should signal the transition between the different phases. We shall discuss this in detail in Sec. *Discussion and Conclusions*.

### Structure

The energy analysis suggests the presence of two clearly distinguishable regimes, i.e. fluid and string gel. However, a detailed examination of the trajectories towards the end of the simulation runs reveals the presence of an intermediate regime, consisting of a distribution of short string-like clusters. This *string-fluid* regime has been identified in previous works^16,17,33^ and presents peculiar structural features. Therefore, we analyzed the system structure, in order to quantify the changes across the different regimes and characterize each of them. We computed radial distribution functions and cumulative radial distribution functions to characterize the structure of the suspension; and mean cluster size, maximum cluster size and cluster-size distributions in order to study the cluster properties.

#### Radial distribution functions

The radial distribution function, *g*(*r*), gives the probability of finding a second particle *j* at distance *r*_*ij*_ from a reference particle *i* relative the complete randomness. Even though this quantity neglects anisotropic effects, it provides important information about the overall structure of the system.

**Figure 4:**
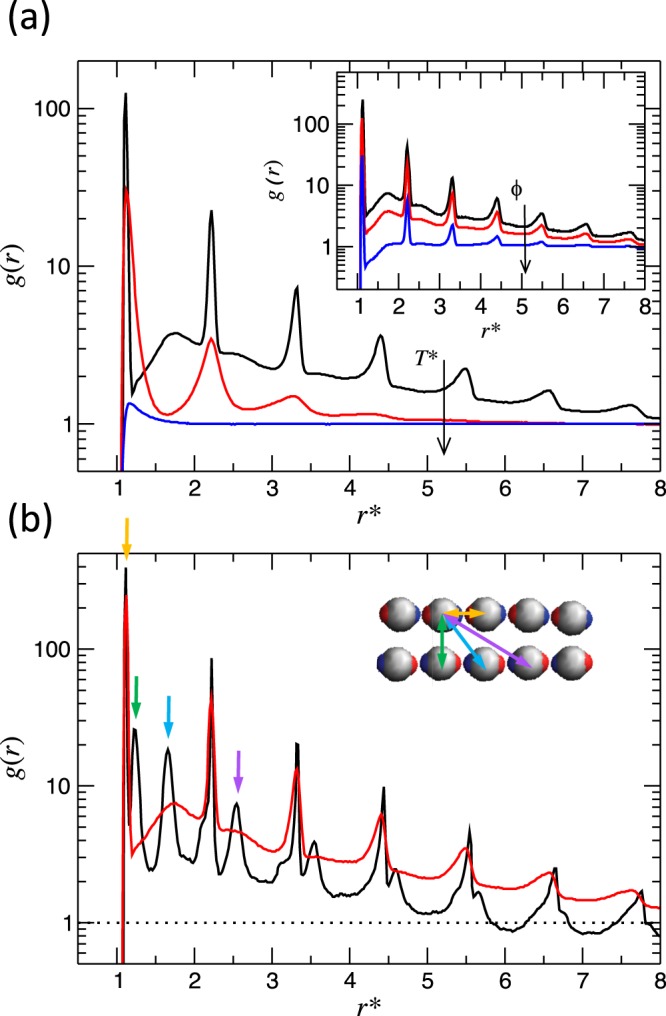
(**a**) Radial distribution function at different temperatures, T^*^ = 0.035 (black); 0.15 (red) and 0.55 (blue), for a fixed volume fraction *ϕ* = 0.012. Inset: Radial distribution function at different volume fractions, *ϕ* = 0.006 (black); 0.012 (red) and 0.049 (blue), for fixed temperature T^*^ = 0.035. (**b**) Radial distribution function at different temperatures, T^*^ = 0.003 (black) and 0.035 (red), for fixed volume fraction *ϕ* = 0.006. The colored arrows indicate the correspondence between the peaks in *g*(*r*) and the colloidal arrangements. Inset generated with VMD^44,45^.

In Fig. 4, we show representative examples of *g*(*r*) for the three mentioned regimes at different temperatures, T^*^, and volume fractions, *ϕ*. The high-temperature example of Fig. 4(a), displayed in blue, corresponds to the fluid regime where the system consists of isolated colloids having a gas-like structure. Decreasing the temperature, we observe the string-fluid regime in red, where the particles associate forming small clusters with short persistence length. The peaks are well localized at distances multiples of *r*_m_, which marks the position of the principal peak and corresponds to the first neighbor distance. This is evidence of linear growth; the colloids aggregate forming mostly linear chains. Decreasing the temperature even further, the system transitions to the string-gel regime, displayed in black on Fig. 4(a). The peaks in *g*(*r*) become sharper and the structure extends over longer distances indicating that the chains become straighter and longer, and the fact that *g*(*r*) approaches one from above for large *r* reveals the presence of density inhomogeneity. The effect of volume fraction on the string-gel regime is displayed in insert of Fig. 4(a). By decreasing volume fraction the colloidal strings become straighter and longer.

In Fig. 4(b) we analyze the effect on the string-gel regime of reaching very low temperatures at a low concentration. We observe that the colloidal particles undergo a more complex association between themselves with further cooling. We also notice the emergence of a secondary structure displayed by the second series of peaks and the faster decays. This secondary structure is associated to the antiparallel alignment of the chains forming bundles, Fig. 1(b). The first two peaks in the secondary structure are associated to the first and second neighbors, respectively, on an adjacent chain. The formation of bundles intensifies the inhomogeneous spatial distribution of the colloids, giving rise to larger void regions. This is reflected at large *r*, where *g*(*r*) is smaller than one. Typically, the splitting of the principal peak is a characteristic for the onset of crystallization. At high dilution and low temperature, we cannot ensure that the analyzed simulations have reached thermodynamic equilibrium. Thus, we can not discuss in the context of thermodynamic phase transition. However, we can discern a sharp peaks following a clear pattern, which indicates locally well-structured bundles.

Following a similar argument to the one of Ilg and Del Gado^22^ and using the electric dipole-dipole interaction energy as approximation, two antiparallel chains of length ≈ 10 colloids experience an overall attraction energy of 0.069 *u*_*d*_ per colloid for a chain separation *σ*. At high temperature, the chains are not long and stiff enough to dominate over the thermal fluctuations. Decreasing temperature and concentration, the chains become straighter and longer, and the thermal energy comes to be comparable to the chain attraction, which favors bundling. From the same calculations, we also observe that for longer chains, the attraction reduces up to 60% for double length chains.

**Figure 5:**
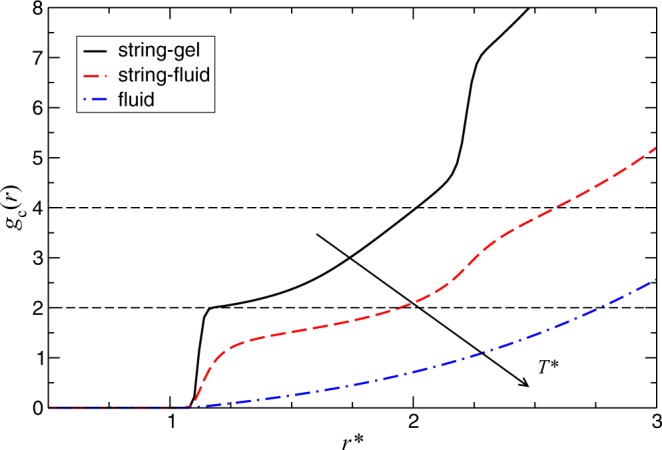
Cumulative radial distribution function for different temperatures for the identified regimes: string-gel, string-fluid and fluid, whose temperatures are T^*^ = 0.035; 0.15 and 0.55, respectively, and volume fraction *ϕ* = 0.012.

Deeper insights in the local structure are obtained by studying the cumulative pair correlation function. The latter is defined in terms of the pair correlation function *g*(*r*) as ${g}_{c}(r)=4\pi (N/V){\int }_{0}^{r}r{\prime} 2g(r{\prime} )dr{\prime}$. In Fig. 5 we show some representative cases. For this particular case of a pseudo-dipolar fluid assembly at low densities, *g*_*c*_(*r*) offers a better understanding of the system structure than *g*(*r*), since it directly provides the average number of neighbors of the central particle disregarding the normalization that it is irrelevant for inhomogeneous systems at long distances. The figure shows one representative example for each one of the three regimes. First, in the fluid regime, the average number of first neighbors is very small, consistent with a fully solvated state. In the string-fluid regime, the formation of strings is indicated by the step-like shape of *g*_*c*_(*r*), with clear steps at separations of one and two particle diameters approximately. The average number of first neighbors is smaller than two, which shows that there is a significant number of short strings. For the string-gel regime, the average number of first neighbors is two, implying that the whole system participates in a percolated string cluster. It is also clearly visible the formation of bundles, as the number of neighbors just above two particle diameters is approximately five, and not four as it should be for a single non-bundled string.

#### Cluster properties

**Figure 6:**
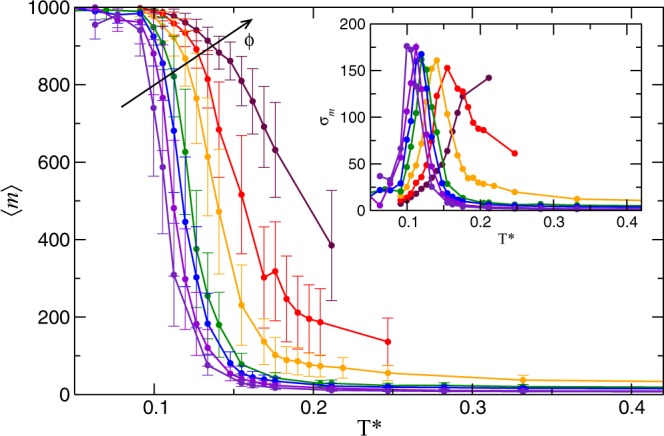
Maximum-cluster size versus temperature for different volume fractions. The error bars are calculated from the standard deviation. Different colors represent different volume fraction, *ϕ* = 0.009; 0.012; 0.016; 0.019; 0.03; 0.039 and 0.049. Inset: Standard deviation of the maximum-cluster size, *σ*_*m*_, versus temperature. The lines are a guide to the eye.

The formation of string-like clusters is the main feature of the string-fluid and string-gel regimes. The string-gel regime is characterized by the formation of a percolating network that can be identified by the size of the largest cluster approaching to the total number of particles in the simulation cell. In Fig. 6, we display the average cluster size $\left\langle m\right\rangle$ versus T^*^ for different volume fractions. $\left\langle m\right\rangle$ is calculated by averaging the last 1000 time steps of the simulation runs. At low and high temperatures, we clearly identify the string-gel and fluid regimes respectively. At low temperature, we observe that the macro cluster practically contains all the particles in the system, particularly at high concentration. The kinetic activity of the macro cluster is reflected in the standard deviation, *σ*_*m*_, which is plotted in the inset of Fig. 6. The noticeable irregularities of *σ*_*m*_ at low concentrations suggest the presence of chains with highly active dead ends, which are more likely at higher dilution. An important aspect, that we have observed through the visualization of the trajectories, is the coexistence of small clusters together with the macrocluster.

As we have described before, the fluid regime is mainly formed by isolated coexisting colloids. Thus, the maximum cluster size tends to one at high temperature in fluid regime. We see that the system clearly describes this behavior for low concentrations, i.e. low volume fraction. The effect of temperature becomes more relevant at higher concentration, leading to a more active process of formation and breaking of small clusters, as indirectly revealed by the larger *σ*_*m*_ and the observation of the trajectories.

**Figure 7:**
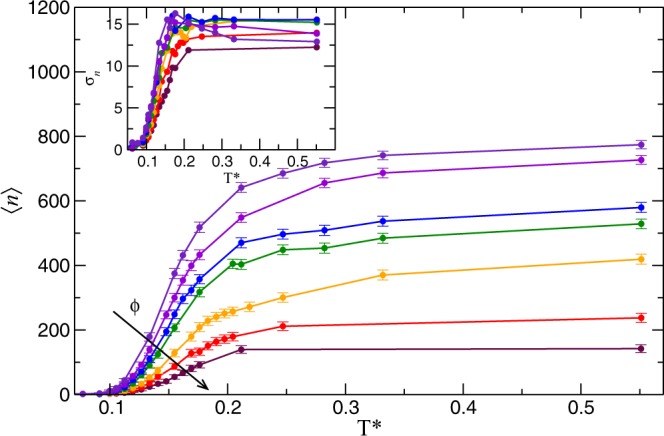
Mean number of clusters versus temperature for different volume fractions. The error bars are given by the standard deviation. Different colors represent different volume fraction and the color code is as in Fig. 6. Inset: Standard deviation of the mean number of clusters, *σ*_*n*_, versus temperature for the same volume fractions. Lines are guide to the eye.

Following the evolution of the largest cluster does not capture the main structural characteristic of either the string-fluid or the fluid regimes, because they are constituted by many small clusters and mostly isolated single particles, respectively. Then, we have studied the mean number of clusters, $\left\langle n\right\rangle$, which is the time-averaged number of different clusters independently of their size. In Fig. 7 we display $\left\langle n\right\rangle$ vs T^*^. For all volume fractions and at low temperature, we have $\left\langle n\right\rangle \lesssim 10$, consistent with the macrocluster regime. Increasing the temperature results in a gradual increase of $\left\langle n\right\rangle$ to reach a plateau at higher T^*^, which is larger as the volume fraction decreases. Notice that for the highest temperature, $\left\langle n\right\rangle  < N$ and therefore there is association between a small number of particles. Having a closer look to *g*(*r*) for these cases, we see that it has the shape of diluted fluid (gas) pair distribution function. Thus, structurally, the system is predominantly formed by isolated colloids. Increasing the concentration of colloids, the *g*(*r*) develops a shallow minimum after the principal peak that we interpret as the onset of short chain formation. We must also notice that for *ϕ* = 0.049 we have ${r}_{{\rm{b}}} > \bar{r}$, with $\bar{r}=a{(4\pi /(3\phi ))}^{1/3}$ being the mean particle distance. Therefore, at higher concentration, the life time of bonds becomes a relevant variable determining the cluster formation. Throughout this work we have not considered the cluster life time as a variable in the bonding criterion, because we are mainly interested in low-concentration/low-temperature regime, where the bonds are highly stable due to the predominance of the electric interaction against thermal fluctuations, *u*_*d*_/*k*_*B*_*T* ≳ 10 .

**Figure 8:**
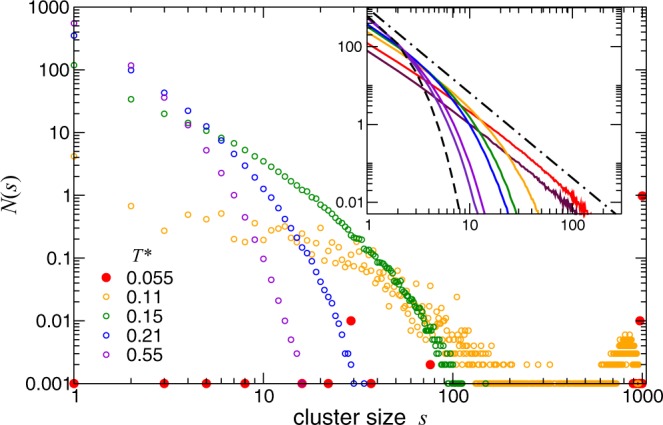
Cluster size distribution for different temperatures at fixed volume fraction *ϕ* = 0.012. Inset: Cluster-size distribution for different volume fractions at constant temperature T^*^ = 0.55, with color as in Fig. 6. The black lines correspond to exponential cluster size distribution (dashed), $N(s)\propto \exp (-cs)$, and power-law cluster size distribution (dash-dotted), *N*(*s*) ∝ *s*^−*τ*^, with *τ* = 2.19^41^.

Further information related to the cluster formation is given by the cluster-size distribution *N*(*s*), which provides the time-averaged number of clusters of size *s*. In Fig. 8, we find *N*(*s*) for a fixed volume fraction at different temperatures. At high temperature, in the fluid regime, *N*(*s*) has a global maximum at *s* = 1 and decays monotonically to zero, namely the system is mostly formed by isolated colloids. By decreasing temperature this maximum decreases and the curve decay with *s* is slower, indicating the assembly of single colloids into new and larger clusters. At low enough temperature, *N*(*s*) develops a peak for large cluster size, showing the emergence of a macrocluster and the string-gel regime. With further decrease of the temperature this peak becomes higher and sharper, indicating a reduction of fluctuations. The number of small clusters is reduced, showing that the macrocluster grows at the expense of the short-string clusters. Notice that decreasing temperature, the data becomes more scattered at small *s*, which also reflects the kinetic activity of the free ends in the formation and breaking of small clusters. Visual inspection of the trajectories reveals that, coexisting with the macrocluster, there are smaller clusters especially at very low temperature. Particularly at low concentration, we find isolated short chains and rings. In the inset of Fig. 8, we observe *N*(*s*) at a fixed (high) temperature for different volume fractions. All the curves show the mentioned monotonic decrease. For better comprehension of the distribution profiles, we also show two typical analytical cases. The first one corresponds to exponential size distribution, $N(s)\propto \exp (-cs)$, which have been experimentally observed for dipolar systems^9^. The second case is a power-law cluster size distribution, *N*(*s*) ∝ *s*^−*τ*^, with *τ* = 2.19 corresponding to random percolation in three dimensions^41^. At high temperature and low volume fraction, we observe that the measured distributions decay approximately, but slower than the exponential case. With increasing volume fraction, the system describe a power-law decay, with exponent slightly smaller than *τ*, indicating the onset of a transition into a percolating regime.

### State diagram

In order to locate the transition between the different regimes we have taken two quantities from the structural analysis as order parameters. Since the maximum-cluster size depicts two clearly different regimes, and the presence of a macrocluster is the main characteristic of the string gel, we have taken this quantity as order parameter to define the transition between string-gel and string-fluid. We take the maximum of its standard deviation at fixed volume fraction as transition temperature. Notice that this transition becomes more abrupt and better defined at low volume fraction, which is in agreement with previous connectivity analysis of similar DHS systems^16^.

The fluid regime corresponds to a gas-like structure in the colloid-colloid pair correlations, while the string-fluid regime is a gas of short chains. Thus, we identify the transition between these two regimes by inspecting *g*(*r*). At given volume fraction, we consider that the system reaches the fluid regime when the second peak of *g*(*r*) vanishes with increasing temperature. This structural feature, together with the fact that the height of the principal peak is very low, indicates that the system is predominantly formed by single colloids. As mentioned before, at the highest temperature, *g*(*r*) shows a weak secondary peak in the most concentrated cases. We have used the cluster size distribution to identify these cases. A different picture emerges from a pure thermodynamic analysis. As shown in Fig. 3(b), the heat capacity shows a single peak separating two phases, suggesting that fluid and string-fluid are either the same phase or there is a continuous transition between them.

**Figure 9:**
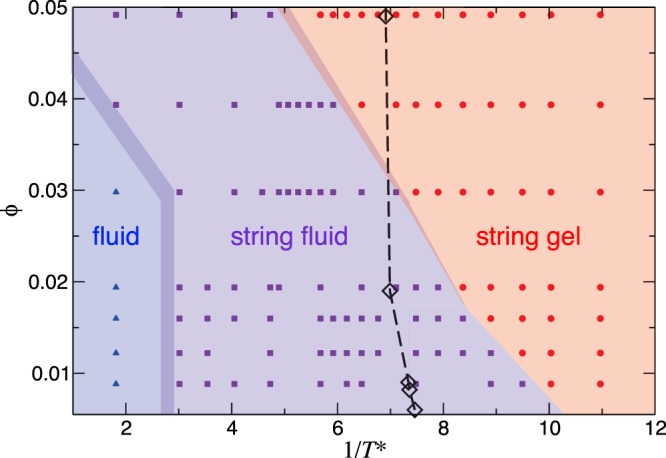
State diagram volume fraction versus temperature. The studied parameter range (T^*^, *ϕ*) is broader than the represented here, but we only show the region where the transitions and remarkable changes take place. The black symbols represent the maximum of the heat capacity, calculated from the fitting function *f*_*U*_(T^*^) shown in Fig. 3(b).

Our results for the state diagram are shown in Fig. 9. Considering purely structural properties as order parameters, the computed state diagram agrees with previous results at larger volume fractions^16^. The transition between fluid and string-fluid regimes does not present remarkable dependence on volume fraction, except for the most concentrated systems. At temperatures close to the transition temperature, the system becomes slightly more structured with increasing concentration, as we have seen from the analysis of *g*(*r*). But these changes are not prominent enough to present noticeable density dependence in the transition temperature within the considered sampling. On another hand, the transition between string-fluid and string-gel shifts to larger temperature as the volume fraction increases, in good agreement with previous connectivity analysis^12^. Consequently, the string-fluid region widens with decreasing volume fraction for diluted systems. This widening arises from the fact that isolated short chains can grow in size, due to the large dilution, without reaching the percolation size. At highly dilute concentrations, in spite of previous uncertainties, short-chain configurations remain stable in the time range that we have studied. The thermodynamic analysis shows a single transition between gel and a fluid phases, indicated also on Fig. 9.

Notice that T^*^ = 0.1 is a typical energy rate for string-gel regime close to the transition for the studied concentrations. It means that the typical coupling energy must be, at least, one order of magnitude larger than the thermal energy for concentrations around 1% in volume in order to find string-gel regime. To find systems with bundle formation, namely antiparallel alignment of the chains, it is necessary to reach energy rates where the typical coupling energy is at least two orders of magnitude larger than the thermal energy. This creates challenging experimental conditions to find the string-gel regime.

## Discussion and Conclusions

The string-fluid regime is essentially formed by kinetically-active small string-like clusters, which cannot grow sufficiently to percolate. We find that its region in the state diagram widens with decreasing concentration: at lower concentration, the regime is formed by longer chains, which are favored by the dilution. The analysis based on structural properties clearly reveals the existence of the string-fluid regime. However, looking at the specific heat we see a single maximum separating a fluid like regime and the string gel. This could mean that: (a) The fluid and string fluid are essentially the same thermodynamic phase or (b) If they are two separate phases the transition between them is a continuous one and likely affected by the finite size of our simulation model.

The string-gel regime is characterized by a permanent macrocluster consisting of a network that extends over the whole system. We have shown how the network structure is affected by overall colloid concentration and temperature. Especially at low temperature, through trajectory analysis and the radial distribution functions, we have shown evidence of the antiparallel alignment of the strings as a novel configurational feature. While at higher concentrations more complex topological features affect the network properties^14^, at high dilution the antiparallel alignment becomes an emerging self-assembly mechanism. Associated with bundling the system will have larger pores, and we expect also an effect on the rheological properties of the gel. The formation of bundles has been reported in similar colloidal systems with permanent dipolar interaction^22^ and, more recently, in microgel systems with induced dipolar interaction^42^, but under the effect of an external field. Here we report for the first time the formation of bundles as one of the assembly mechanisms in the low-density low-temperature limit and with no external field and particles with isotropic short-range repulsion. It is also important to note that experimental results confirming 2D low-concentration string-gel formation^9,40^ were performed on energy ranges similar to the ones that we explored. These experiments also found similar string-like structures to the ones obtained with our model.

**Figure 10:**
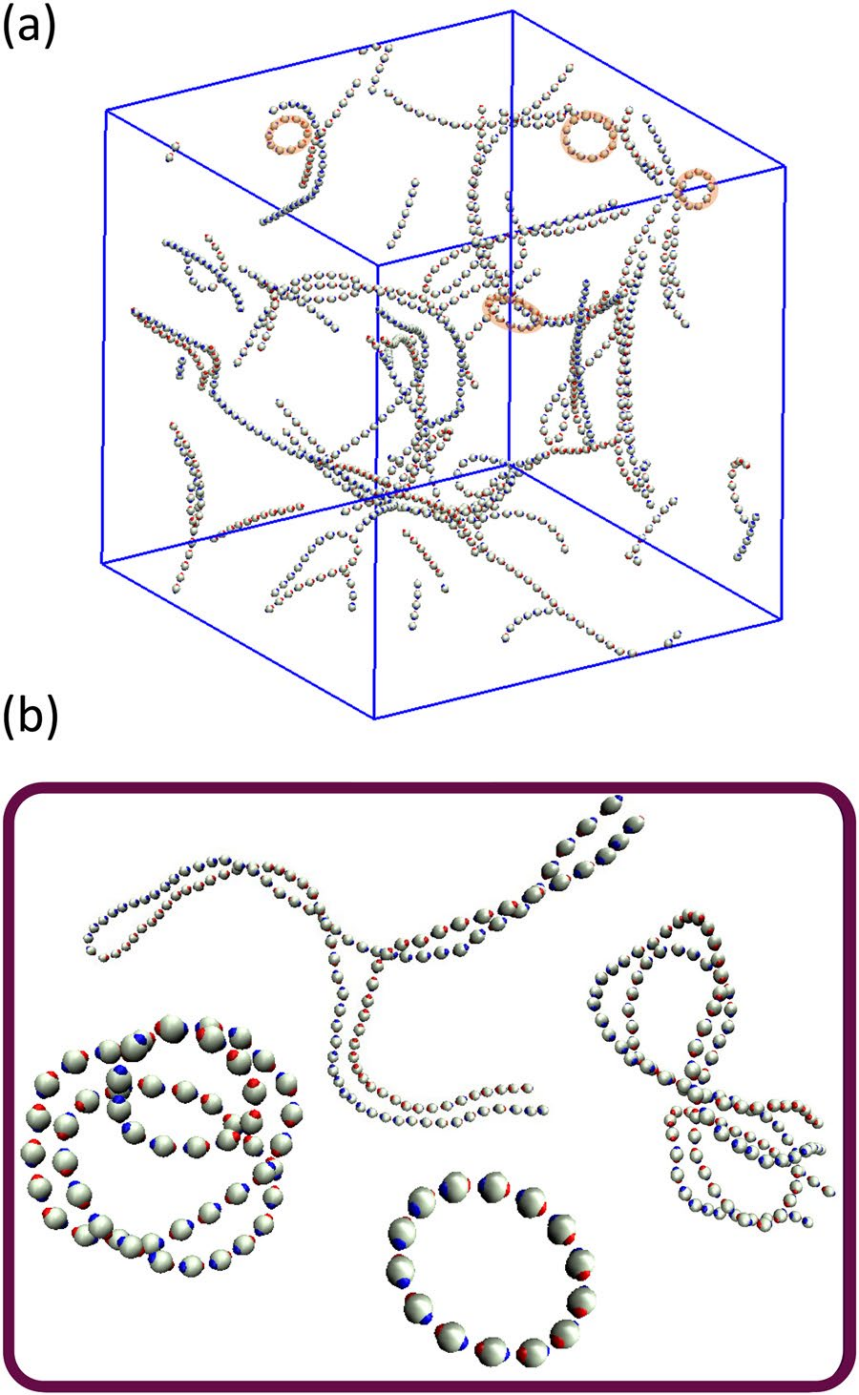
(**a**) Representative example of a string-gel configuration at high dilution, *ϕ* = 0.006 and T^*^ = 0.0035, where ring-like clusters appear. (**b**) Examples of different cluster structures favored by emergence of different topological defects. Figures (**a**,**b**) generated with VMD^44,45^.

From the analysis of the particle trajectories, we highlight the formation of ring-like clusters at very low concentrations as another relevant structural feature. From previous works, we know that they are weakly interacting objects and deprive the fluid phase of chain ends, which were predicted to sustain the topological phase separation^8,11–13^, namely the transition between string-fluid and string-gel regimes in our context. We have found ring-like clusters coexisting with the macrocluster network or bound to its bundles at low concentration. In Fig. 10(a), we show a representative example of a configuration with ring-like clusters. Due to the high dilution, the rings turn out to be stable arrangements that reduce the energetically-expensive chain ends.

The tail-nose assembly, the antiparallel alignment and the ring formation are combined with different topological defects in order to give rise to a wide variety of cluster geometries. The most frequent topological defects that we have found are: triple points or Y-shaped junctions, Fig. 1(b), which favor the chain bifurcation and are very important for the macrocluster percolation^8^; and the quadruple points X-shaped junctions, which favor the crossing of two nonparallel strings. In Fig. 10(b), we find examples of these complex clusters. We have also found short strings coexisting with the macrocluster. These coexisting chains are too short to be able to bend and form rings. Because these chains have free ends, this type of configurations are unstable although they may have a long lifetime.

**Figure 11:**
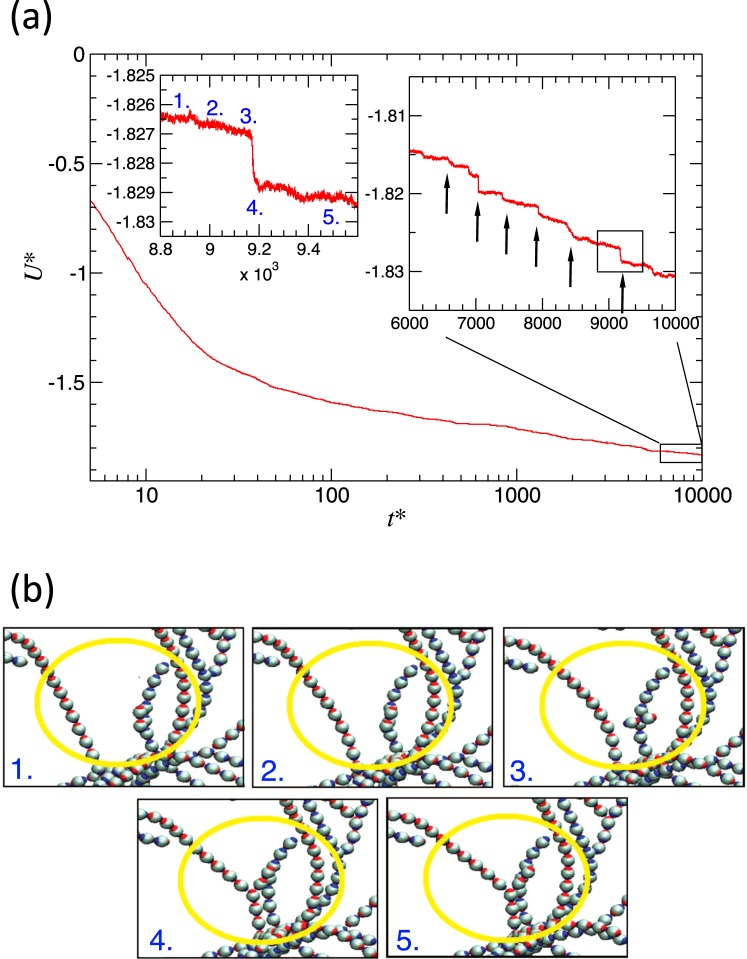
(**a**) Potential energy versus time for *ϕ* = 0.0083 and T^*^ = 8.0 × 10^−5^. The insets represent zoom of the selected regions. The arrows highlight the energy drops in steps. (**b**) Snapshot sequence corresponding structural changes associated to the energy drop in one step. Snapshots generated with VMD^44,45^.

In order to gain insights on the time evolution of a string-gel system at low concentration and very low temperature we performed extended simulation runs for particular cases. In the Fig. 11(a) we see the time evolution of the potential energy for one of these extended simulations. It can be observed that the energy suffers changes of around 1% in the last half of decade. A closer look reveals that the potential energy decreases in clear steps and each step is associated to rearrangements of the network, i.e. by breaking and bonding chains, producing small local reconfiguration of the macrocluster, Fig. 11(b). In this way, the system slowly relaxes to a more energetically favorable structure. As we observe in the insets of Fig. 11(a), each energy step is in the order of ΔU^*^ = 0.002. Considering that there are 1000 particles, the energy change is associated to the rearrangement of just a few colloids. Experimental findings on similar systems at comparable thermodynamic conditions display characteristics of a dynamic arrest^19,20^. Our simulations are in line with those findings.

Our results are summarized in the state diagram of Fig. 9, which shows the three regimes already described. From thermodynamics analysis, we find that there is a single phase transition separating the string-gel and a fluid phase. However, experimental findings have characterized a transition between a string-fluid and string-gel at low concentrations for 1/T^*^ between 4 and 9^9^, in agreement with our findings based on configurational analysis. Considering that experiments also report an arrested dynamics, we may speculate that the experimental system cannot reach its thermodynamic equilibrium and the state diagram is an effective phase diagram for the system.

The experimentally-found structures were achieved in 2D systems of magnetite nanoparticles of *σ* ≈ 20 and 24 nm^9^, consisting of single-domain magnetite crystals. This means that the electrostatic potential of the dipole-dipole assembly is practically one order of magnitude larger than the thermal energy, for string-like structures. According to our findings, the antiparallel alignment can be found at 1/T^*^ ≈ 300 (outside the state diagram shown in Fig. 9). These are very challenging experimental as well as synthesis conditions. Considering that the magnetic moment grows proportionally to the magnetic volume^40^, magnetite nanoparticles of *σ* ≈ 76 nm^43^ would provide an effective interaction strength corresponding to 1/T^*^ ≈ 400, with a particle coating of 2 nm.
